# Tannins Enriched Fraction of *Emblica officinalis* Fruits Alleviates High-Salt and Cholesterol Diet-Induced Cognitive Impairment in Rats via Nrf2–ARE Pathway

**DOI:** 10.3389/fphar.2018.00023

**Published:** 2018-01-30

**Authors:** Ibraheem Husain, Mohd Akhtar, Tushar Madaan, Divya Vohora, Malik Z. Abdin, Mohammad Islamuddin, Abul K. Najmi

**Affiliations:** ^1^Department of Pharmacology, School of Pharmaceutical Education and Research, Jamia Hamdard University, New Delhi, India; ^2^Department of Biotechnology, School of Chemical and Life Sciences, Jamia Hamdard University, New Delhi, India

**Keywords:** cognitive impairment, *Emblica officinalis*, high-salt and cholesterol diet, oxidative stress, Nrf2, Alzheimer’s disease, dementia

## Abstract

Modern diets containing high quantities of salt and cholesterol have exhibited to cause a considerable effect on our health. Such diets, when consumed in the long term, have also shown to be a precursor to several disorders such as the metabolic disorder and consequently, various other diseases, including cognitive deficits. In the present study, we used a high salt and cholesterol diet (HSCD) to induce cognitive impairment in rats and also investigated the pharmacological action of tannins enriched fractions of *Emblica officinalis* (EOT) – a fruit that has been traditionally used for the treatment of numerous disorders for centuries. Significant alterations in MDA, GSH, TBARS, GPx, mitochondrial ATP, and mitochondrial membrane potential levels were observed in rats fed HSCD, which indicated presence of oxidative stress. Moreover, classic signs of cognitive impairment and deficits in spatial learning and memory were observed in the neurobehavioral tests. *E. officinalis* tannins exhibited good affinity to Nrf2 receptors in *in silico* studies, significantly reversed the changes in the aforementioned biomarkers of oxidative stress which were altered in the model group, as well as improved the performance of rats in Morris water maze task. Our results also reflected that EOT supplementation significantly increased the expression of Nrf2 in the CA1 region of hippocampus and cortex. Additionally, TUNEL assay indicated that EOT supplementation led to reversal of DNA fragmentation and apoptosis caused by HSCD. Immunohistochemical analysis and western blot further revealed a surge in the nuclear location of Nrf2. Through our study, we have demonstrated that cognitive impairment can be caused in rats via HSCD as a result of the oxidative stress induced by the same. Additionally, we have investigated a novel mechanism of action for EOT (which strongly suggests to be via the Nrf2–ARE pathway) and demonstrated that it has the potential to be used in the treatment of cognitive impairment.

## Introduction

Dementia is a clinical syndrome characterized by a progressive decline in memory in addition to impairment or difficulties in cognitive functions such as language (aphasia), executive function (such as abstract reasoning, planning, attention), or skilled movements (apraxia). It is estimated that about 135 million people would suffer from dementia by the year 2050 and that the global cost of dementia care would increase to one trillion United States dollars by the year 2030 (American Psychiatric Association, 2013; Prince et al., 2014). Dementia is one of the major causes of morbidity, dependence, and disability in the world and this has led to a global focus toward research related to its early diagnosis and treatment. Alzheimer’s disease and vascular dementia are the two most common forms of dementia (Scott and Barrett, 2007). Numerous risk factors that have shown to increase the susceptibility of a person to suffer from dementia. Among psychological factors – depression, anxiety, sleep disorders and mental distress in mid or late life have shown to influence risk of dementia. Similarly, as far as lifestyle and cardiovascular risk factors are concerned smoking, hypertension, obesity, hyperlipidemia, and diabetes have shown to play a role in the pathogenesis of dementia (Tzourio, 2007).

Diets containing high amounts of salt and cholesterol (a precursor to the aforementioned risk factors) have been implicated in causing cognitive impairment (Husain et al., 2017b). One hypothesis correlates that high salt consumption and cognitive decline are associated with hypertension as well as the consequent white matter lesions, which have been observed in dementia patients. However, solitary effect of high sodium intake (without the presence of hypertension) has also been associated with cognitive decline. There are other hypotheses that connect high sodium intake and diminution in cognition such as loss of integrity of blood–brain barrier due to increased sodium intake or hypothalamic paraventricular nucleus (PVN) function (Fiocco et al., 2012). Similarly, diets containing high quantities of cholesterol and saturated fats have also been linked to cognitive deficit (Kim et al., 2017). There are numerous schools of thought about the mechanism of action underlying cognitive decline and dementia associated with a high-fat diet. One of the prominent hypotheses considers oxidative stress caused by consumption of such diets to be the culprit of causing cognitive impairment. Other hypotheses implicate neuroinflammation, insulin resistance, and dysfunctional vascularization for it (Granholm et al., 2008). Hence, in this study, we have employed a high salt and cholesterol diet (HSCD) to induce cognitive impairment in the rats, as it is a natural and practical method since it mimics natural pathogenesis.

Increased oxidative stress is a consistent feature in various forms of dementia. Significant damage due to oxidative stress is also commonly seen in peripheral blood during the neurodegeneration process (García-Blanco et al., 2017). Oxidative damage to proteins, lipids, DNA/RNA has also been observed in human autopsy brain tissues (Head and Zicker, 2011). Significantly higher quantities of lipid peroxidation biomarkers have been observed in plasma, urine, and cerebrospinal fluids of subjects suffering from mild cognitive impairment (Praticò et al., 2002). Evidence of abundant protein nitration and nucleic acid oxidation has also been noted. In fact, studies have also indicated that those subjects who have low antioxidant status are relatively more vulnerable to cognitive deficit and dementia than corresponding subjects with relatively higher antioxidant status (Baierle et al., 2015). There is an endogenous pathway in our body, which helps to mitigate increase in cellular oxidative stress. This mechanism, which is regulated at the transcriptional level, also helps to minimize accumulation of toxic metabolites and inflammation in addition to controlling oxidative stress. A promoter element known as the electrophilic response element or the antioxidant response element (ARE) is found common to genes whose transcriptional products are involved in the aforesaid activities. Multiple transcription factors are bound to ARE, but the nuclear factor (erythroid-derived 2)-like 2 (Nrf2) plays a crucial role in controlling oxidative stress by activating transcription when oxidative stress reaches abnormal levels (Itoh et al., 1997). Nrf2 has shown neuroprotective activity in numerous studies. Moreover, inhibition of Nrf2 inhibitors/suppressors has also shown to alleviate cognitive impairment (Kerr et al., 2017). In fact, reduction in Nrf2 expression is commonly observed in Alzheimer’s patients (Kanninen et al., 2009). Hence, the Nrf2-ARE pathway has been considered to be a promising therapeutic target for the treatment of various forms of dementia.

Recent research studies have underscored the potential role of the Nrf2-ARE pathway in numerous neurodegenerative disorders. As mentioned earlier, decrease in Nrf2 in the brains of Alzheimer’s patients is a common feature and is considered to be the possible reason behind the increased vulnerability of neurons to stresses in these patients (Ramsey et al., 2007). Most of the presently approved therapies for neurodegenerative disorders such as Alzheimer’s disease (donepezil, galantamine, rivastigmine, memantine) and Parkinson’s disease (L-DOPA and MAO-B inhibitors) only compensate for the damage already caused, and show their pharmacological activity by regulating neuronal activity and acting on receptors or affecting neurotransmitters (National Institute for Health and Clinical Excellence, 2010). These therapies do not tackle the underlying pathophysiologies that cause neuronal death, such as mitochondrial dysfunction and oxidative stress – which are considered as contributing factors to these diseases. Therefore, there is an imminent need to investigate novel therapeutic options to address these issues.

Several herbal drugs have been used traditionally for innumerable years for the treatment of cognitive impairment. *Emblica officinalis* Gaertn. (Amla or Indian gooseberry) is a plant, which grows in the tropical regions of India, Indonesia, China, and the Malay Peninsula. It is a member of the Euphorbiaceae family, and the fruit of this plant has traditionally been used for the treatment of a multitude of ailments in the Ayurveda, Unani, Tibetan, Siddha, Arabic and various other folk systems of medicine. The fruit contains several tannins such as emblicanin A, emblicanin B, pedunculagin, punigluconin, gallic acid, rutin, etc. In a study by Bhattacharya A. et al. (2000), it was observed that tannins enriched fractions of *E. officinalis* (EOT) fruits normalized alterations in frontal cortical and striatal superoxide dismutase, glutathione peroxidase, catalase, and lipid peroxidation induced by chronic stress via chronic unpredictable foot-shock induced perturbations. In another study, EOT reversed amnesia caused due to scopolamine administration as well as reduced oxidative stress induced by it. In addition, it also reversed the increased acetylcholinesterase (AchE) levels in the brain (Golechha et al., 2012). EOT has also shown protective activity against neurotoxicity induced by aluminum (Justin Thenmozhi et al., 2016). The antioxidative properties of EOT are considered to be responsible for their activity against a variety of oxidative stress-induced neurological disorders such as tardive dyskinesia, alcohol-induced brain mitochondrial function, etc. (Bhattacharya S.K. et al., 2000). Recent studies have investigated various potential mechanisms of action of EOT such as suppression of amyloid precursor protein, beta-secretase, and gamma-secretase as well as a potential mechanism via the Akt/GSK-3β pathway (Thenmozhi et al., 2015, 2016). However, since the pathophysiologies of most neurobehavioral disorders are multifactorial, it is essential to explore novel potential mechanisms. In this study, we have, for the first time, demonstrated a possible pharmacodynamic action of EOT via the Nrf2 pathway.

## Materials and Methods

### Drugs and Chemicals

Tannins enriched fractions of *E. officinalis* (EOT) fruits were provided as gift samples by Indian Herbs Research and Supply Company (Saharanpur, India). The tannins were extracted as per the method given by Ghosal et al. (1996) which involved deactivating the hydrolytic enzymes present in the fresh juice of *E. officinalis*, following which, column chromatography was performed over Sephadex LH-20 employing methanol and methanol-water as the eluent. The concentration of the various tannins was as follows: emblicanin A (37%), emblicanin B (33%), punigluconin (12%), pedunculagin (14%), rutin (3%), and gallic acid (1%) (Ghosal et al., 1996). Piracetam (PCT) was provided as gift sample by Arbro Pharmaceuticals Ltd. (Delhi, India). ATP bioluminescence assay kit and JC-1 (5, 5, 6, 6- tetrachloro-1,1,3,3- tetraethyl benzimidazolyl carbocyanine iodide) were obtained from Sigma–Aldrich (India). BCA protein assay kit was procured from Span Diagnostics Limited, Gujarat, India. Annexin V– fluorescein isothiocyanate (FITC) and ApoDirect kits were purchased from Roche Inc. All other reagents used in the experiments were of analytical grade. Double-distilled water was used throughout the experimental work.

### Animal Procurement

The experimental protocol was approved by Institutional Animal Ethics Committee of Jamia Hamdard (Hamdard University), New Delhi, India (Registration no. JH/993/CPCSEA) as per the guidelines of Committee for the Purpose of Control and Supervision of Experiments on Animals. Female Wistar rats in the range of 150–200 gm of body weight were issued from Central Animal House Facility, Jamia Hamdard, New Delhi and housed in standard polypropylene cages (six rats each cage) and had access to commercial standard pellet diet (Amrut rat feed, Nav Maharashtra Chakan Oil Mills Ltd., New Delhi, India). The rats were maintained under controlled room temperature (23 ± 2°C) and relative humidity (60 ± 5%) with 12 h light/12 h dark (day/night) cycle in the departmental animal house.

### Preparation

Suspension of EOT and PCT were prepared by triturating the weighed amount of EOT (100 and 200 mg/kg) and PCT (200 mg/kg) in 0.5% carboxy methyl cellulose (CMC) suspension (w/v) in normal saline, respectively (Ghaisas et al., 2010). High salt saline was prepared freshly by adding 2% w/v NaCl in water. Pellets containing high cholesterol were prepared freshly by adding 1.25% cholesterol and 10% coconut oil in standard diet pellets and dried at room temperature.

### Experimental Design

**FIGURE 1:**
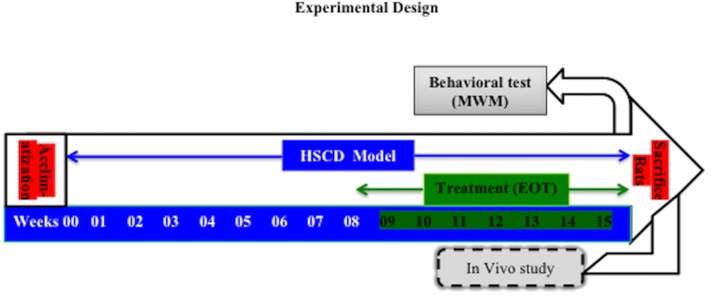
The experimental design of the study. HSCD, high-salt and cholesterol diet; MWM test, Moris water maze test; EOT, Tannins enriched fraction of *Emblica officinalis*.

Prior to the commencement of experimental studies, animals were fed with standard rat food pellets for 2 days. Then, animals were fed a HSCD *ad libitum* for 8 weeks to induce cognitive impairment (Mogi et al., 2007; Husain et al., 2017a). After that, rats were treated with EOT (p.o.), PCT (i.p.) for 7 weeks in different doses (**Table 1**). The rats were observed for behavioral parameters (Moris water maze test) and then immediately sacrificed for histopathological examination and estimation of biochemical parameters (**Figure 1**).

**Table 1:** Different experimental groups and treatment conditions.

Group treatment (*n* = 6)	Treatment
NC (normal control)	NS and ND^a^
TC (toxic control)	HSCD^a^
EOT100	HSCD^a^ and EOT (100 mg/kg b.wt)^b^
EOT200	HSCD^a^ and EOT (200 mg/kg b.wt)^b^
PCT200	HSCD^a^ and PCT (200 mg/kg b.wt)^b^

*n, number of rats in each group; HSCD, high salt and cholesterol diet; NS, normal saline; ND, normal diet; EOT, tannoid principles of *Emblica officinalis*; PCT, piracetam. ^*a*^Administered once daily from 1st to 105th day. ^*b*^Administered once daily from 57th to 105th day*.

### Molecular Docking Analysis

In molecular docking, *in silico* studies were performed to evaluate the predominant binding mode as well as the binding affinity of the chief EOT constituents with the target receptor (Morris and Lim-Wilby, 2008). Maestro 10.5 program (Schrodinger Inc., United States) was used to predict binding modes of ligands into Nrf2-DNA complexes receptor pockets. The Nrf2-DNA complexes is a validated target for cognitive impairment and the three-dimensional structure of Nrf2-DNA complexes was downloaded from protein data bank (PDB code: 2FLU) (Magesh et al., 2012). The various steps involved in the molecular docking studies are selection and preparation of appropriate protein, grid generation, ligand preparation and then docking and its analysis. The protein preparation was performed in three steps, i.e., preprocess, review and modify and refinement using ‘protein preparation wizard’ in Maestro 10.5. Water molecules were deleted and hydrogen atoms were added in these steps. OPLS 2005 force field was used to reduce the energy of the ligands. A default box (receptor grid generation) was prepared by clicking the most appropriate site shown by running site map program. The ligand was docked into the grid generated from the protein using extra precision (XP) and the results were evaluated by glide score. The docking score, hydrogen bonds and pi–pi interaction formed with the surrounding amino acids are used to predict theoretical binding affinities and proper alignment of the molecules at the active site of the Nrf2-DNA.

### Morris Water Maze Test

Morris water maze test – a commonly used test to evaluate spatial learning and memory in rodents – was used to perform neurobehavioral assessment (Bromley-Brits et al., 2011). The experimental apparatus consisted of a black circular water pool with diameter 1.50 m and height 0.60 m. The water inside the pool was maintained at a temperature of 24 ± 2°C. The apparatus had a featureless inner surface and divided into four equally spaced quadrants (NE, SE, NW, SW). A translucent 10 cm × 10 cm platform, submerged 1 cm below the water surface, was hidden in the center of quadrant NE (target quadrant) during the training period and was then removed at the time of the probe task. The training was conducted four times a day for five consecutive days before the probe task. Each rat was allowed to swim until they found the platform or until 120 s elapsed. Then, the rat was left on the platform for 10 s. During the spatial probe task, the platform was removed from the pool, and the rats were allowed to swim for 120 s. The swim escape latency, path length and time spent in the target quadrant were recorded by a video tracking system (SMART v3.0.03 software, Panlab Harvard, United States).

### Measurement of GSH, GPx, and MDA

Reduced glutathione (GSH) was estimated using the colorimetric method as described by Ellman (1959). Equal quantities of brain homogenate (w/v) and 10% trichloroacetic acid were mixed and centrifuged at 3000 rpm for 15 min. 2 mL of phosphate buffer (pH 7.4), 0.5 mL 5,5-dithiobisnitro benzoic acid (DTNB) and 0.4 mL of double-distilled water were added to 0.01 mL of supernatant of the above mixture. Then, the mixture was vortexed, and their absorbance was recorded at 412 nm within 15 min of the addition of DTNB.

Glutathione peroxidase (GPx) activity in brain was assayed spectrophotometrically through the glutathione/NADPH/GR system, by the dismutation of H_2_O_2_ at 340 nm (Hassan et al., 2015). GPx activity is measured indirectly via this method using NADPH disappearance. The enzymatic activity was expressed in nmol NADPH min^-1^ mg^-1^ protein.

The thiobarbituric acid reactive substances (TBARS) test was used to determine malondialdehyde (MDA) – a biochemical produced during the lipid peroxidation process (Utley et al., 1967). 0.1 mL of brain homogenate was pipetted into a 13 mm × 100 mm test tube and incubated at 37°C in a metabolic shaker for 1 h. An equal volume of homogenate was pipetted into a centrifuge tube and placed at 0°C and marked at 0 h incubation. After 1 h of incubation, 0.45 mL of 5% (w/v) chilled TCA and 0.45 mL 0.67% thiobarbituric acid was added to the above homogenate and centrifuged at 4000 × *g* for 10 min. Thereafter, supernatant was transferred to other test tubes and placed in a boiling water bath for 10 min. The absorbance of pink color produced was measured spectrophotometrically at 535 nm. The TBARS content was calculated by using a molar extinction coefficient of 1.56 × 105 M^-1^ cm^-1^ and expressed as nmol of TBARS formed/hr/mg of protein.

### Measurements of Membrane Potential, ROS, and ATP Level

Preparation of Isolated Mitochondria: The rats were euthanized with isoflurane after completion of behavioral studies and the procedure given by Dragicevic et al. (2010) was used to isolate the brain mitochondria. After prompt removal, the brains were placed on ice, following which, the hippocampi (*n* = 6) were carefully dissected as per anatomical guidelines and placed in a glass Dounce homogenizer containing five times the volume of isolation buffer (215 mM mannitol, 75 mM sucrose, 0.1% BSA, 1 mM EGTA, 20 mM HEPES, pH 7.2). A low-speed spin (1300 g for 5 min) was performed to remove unbroken cells and nuclei after the homogenization process. The supernatant was transferred to fresh tubes, topped off with isolation buffer and spun down again at 13,000 × *g* for 10 min. The supernatant was discarded, and the resultant mitochondrial pellets were suspended in 500 μl of isolation buffer with 1 mM EGTA (215 mM mannitol, 75 mM sucrose, 0.1% BSA, 20 mM HEPES, pH 7.2) and 0.1% digitonin (in DMSO) was added to the pellets to disrupt the synaptosomes. After 5 min, isolation buffer containing 1 mM EGTA was used to bring the samples to a final volume of 2 mL, after which it was centrifuged at 13,000 × *g* for 15 min. Next the pellets were resuspended in isolation buffer without EGTA (75 mM sucrose, 215 mM mannitol, 0.1% BSA, and 20 mM HEPES with the pH adjusted to 7.2 using KOH) and was centrifuged at 10,000 × *g* for 10 min. Isolation buffer without EGTA was used to suspend the final mitochondrial pellet to yield a final protein concentration of approximately 10 mg/mL and was immediately stored on ice. In order to normalize the results, the concentrations of protein were estimated with all the samples on the same micro well plate using a BCA protein assay kit.

### Membrane Potential Measurements

A 200 μM stock solution of JC-1 (5, 5, 6, 6- tetrachloro-1,1,3,3- tetraethyl benzimidazolyl carbocyanine iodide) was made employing dimethyl sulfoxide (DMSO) as the solvent. The assay buffer consisted of mitochondrial isolation buffer along with 5 mM pyruvate and 5 mM malate. 150 μl of assay buffer and 20 μl (1.2 mg/mL final concentration) of mitochondria were added to the wells of a 96-well black, clear bottom microplate (Corning). 1 μM JC-1 was added and gently stirred. Aluminum foil was used to cover the microplate, and the microplate was left for 20 min at room temperature before observation. Fluorescence (excitation 530/25 nm, emission 590/35 nm) was then measured (Ly et al., 2003).

### Reactive Oxygen Species (ROS) Measurements

Production from isolated mitochondria, Mitochondrial ROS production was measured following incubation of isolated mitochondria with 25 μM 2,7- dichlorodihydrofluorescein diacetate for 20 min and then the DCF fluorescence (excitation filter 485/20 nm, emission filter 528/20 nm) was read as previously described (Brand and Esteves, 2005). In short, 100 μg (0.8 mg/mL final concentration) of isolated mitochondria were added to 120 μl of KCl-based respiration buffer with 5 mM pyruvate and 2.5 mM malate added as respiratory substrates and 25 μM 2,7-dichlorodihydrofluorescein diacetate. Mitochondrial ROS production in the presence of oligomycin (to increase ROS production) or FCCP (to decrease ROS production) was performed to ensure measurement values were within the range of the indicator.

### ATP Level Determination

Luminometric method [using ATP bioluminescence assay kit (Sigma, St. Louis, MO, United States)] was used to determine ATP levels according to the provided protocol. Mitochondrial samples were assayed for ATP content using the ATP dependence of the light emitting luciferase-catalyzed oxidation of luciferin. ATP concentration was calculated according to a standard curve and related to protein content (Wang et al., 2014).

### Immunohistochemical Analysis of Nrf2 Protein

For immunohistochemical analysis of Nrf2 protein, paraffin sections of the brains were deparaffinized in xylene followed with acetone for 5 min each. A graded series of ethanol was used to rehydrate the samples. The samples were then washed with double distilled water, after which, antigen retrieval was performed by citrate buffer (pH 6). Three changes of section were done with TBS buffer solution. These sections were then blocked with 1.5% normal goat serum for 1 h. Sections were then incubated overnight at 4°C with purified goat polyclonal antibody raised against a peptide mapping at the N-terminus of Nrf2 of human origin (1:200; Santa Cruz Biotechnology). Biotinylated anti-goat rabbit secondary antibodies and avidin-biotin-peroxidase complex was used to detect immunoreactivity. Immunoreactive signal was developed using diaminobenzidine as a substrate for 2 min. A Meiji microscope enabled with lumenera camera was used to take the photomicrographs. The images were analyzed with lumenera analyze 3 software. All immunohistochemical samples were analyzed in a blinded fashion. For the quantification of the protein expression semi automatically, Image J 1.49 software was used to estimate the volume fraction of immune-reactive cells within the tissue sample. The range of pixel intensities of images was in between 0 and 250. Values 0 and 250 indicates the darkest and lightest shade of the image color, respectively (Nguyen et al., 2013).

### Western Blotting

Frozen brain was homogenized with 15 volumes (w/v) of TBS buffer, containing phosphatase and protease inhibitor cocktails (Sigma, United States), followed by centrifugation at 100,000 *g* for 1 h at 4°C. The soluble fraction was collected as the TBS-soluble fraction, and the pellet was resuspended in 15 volumes of 1% Triton X-100/TBS (TBSX). After incubation for 30 min on ice, the tube was centrifuged at 100,000 *g* for 1 h at 4°C. The supernatant was removed as the TBSX-soluble fraction, and 70% formic acid (FA) was added to the pellet, followed by centrifugation at 100,000 *g* at 4°C for 1 h. The FA fraction was neutralized with 20 volumes 1 M Tris-base (pH 11), and subsequently aliquoted and stored at -80°C. The total protein level of TBS-, TBSX-, and FA- was determined using the BCA protein assay kit (Bioworld, United States) and the Bradford protein assay kit (Bioworld, United States). For western blotting, equal quantities of protein extracts were subjected to SDS-PAGE and transferred to polyvinylidene difluoride (PVDF) membranes (Millipore, United States) (Chan et al., 2017). After blocking in 5% non-fat milk for 1 h at room temperature, the membranes were incubated overnight at 4°C with the following primary antibody anti –Nrf2 (1:1000, BioLegend, United States). After washing with TBST, the membranes were incubated with HRP-conjugated secondary antibodies for 2 h at room temperature. The signals were visualized using an ECL kit (Bioworld, United States), and the band intensities were detected using Image J software.

### Terminal Deoxyribonucleotidyl Transferase (TdT)-Mediated dUTP Nick-End Labeling (TUNEL) Assay

TUNEL assay, which is used to detect the apoptosis, was performed according to the manufacturer’s instructions (Roche Inc.) (Shadab et al., 2017). Briefly, 500 μl of brain cells sample was harvested by centrifugation and washed twice with 1 mL of PBS. The cells were then resuspended in 0.5 mL of PBS and fixed by adding 5 mL of fresh, prechilled 1% formaldehyde/PBS and incubated at 4°C for 20 min. The above step was repeated one more time. Resuspended cells in 0.5 mL of PBS were then permeabilized by adding 5 mL of 70% ice-cold ethanol and incubated at -20°C for at least 4 h. The cells were washed twice in PBS and then transferred to an amber 1.5 mL microcentrifuge tube (to protect samples from light). The cells were then resuspended in 80 μL of equilibration buffer and incubated at room temperature for 5 min. The cells were washed with PBS and resuspended in 50 μl of TdT incubation buffer and incubated at 37°C in a water bath for 60 min, protected from direct light. Then 1 mL of 20 mM EDTA was added to terminate the reaction, and mixed by gentle vortexing. The cells were pelleted down by centrifugation and resuspended in 1 mL of 0.1% Triton X-100/BSA/PBS. The cells were then resuspended gently in 0.5 mL of PI/RNase/PBS to stain with PI following incubation at room temperature in the dark for 15–30 min. They were analyzed on a flow cytometer, Becton Dickson Canto II with BD FACS Scan Software (San Diego, CA, United States).

### Statistical Analysis

Results were expressed as the mean ± standard error of mean (SEM). The statistical significance of difference between groups was determined using one-way analysis of variance (ANOVA) followed by Tukey’s test. *P*-value < 0.05 was considered statistically significant. Error bars represent the standard error of the mean (SEM). All statistical tests were performed using the Prism software package (version 4, GraphPad, San Diego, CA, United States).

## Results

### Molecular Docking Analysis

**FIGURE 2:**
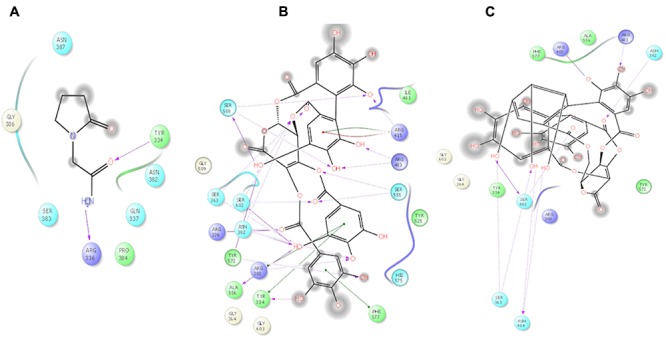
2D ligand interactions of Piracetam and EOT (Emblicanin A and Emblicanin B). **(A)** Piracetam, **(B)** Emblicanin A, **(C)** Emblicanin B showing hydrogen bond interaction with purple color arrow line and pi–pi stacking with green line in the binding site of nrf2.

The theoretical affinity of *E. officinalis* tannins and piracetam were investigated through docking study using Schrodinger programme Maestro 10.5. The ligands were docked into the active site of Nrf2 binding domain (PDB 2FLU) generated by running the site map program and free binding energy, docking scores, interacting amino acid residues H-bonds and stacking amino acid residues pi–pi bond were determined. Emblicanin A – one of the chief constituents of EOT – was well located inside the binding site and the results had revealed that emblicanin hydroxyl and keto group interact in the Nrf2 binding sites with the amino acid Ser508, Arg415, Arg483, Ser555, Ser363, Arg336, Tyr572, Arg380, Tyr334, Ala556. The standard drug piracetam showed two hydrogen bonds, i.e., Tyr334 and Arg336 with the keto and amine group present in the drug similar to the ligand emblicanin. Furthermore, hydrophobic interaction was observed with the amino acids Ile461, Tyr525, Hie575, Phe577, Gly603, Gly364, and Gly509 with emblicanin A. The more hydrogen as well as hydrophobic binding with the drug emblicanin A as compared to the standard piracetam resulted in higher docking score. Other tannins such as punigluconin and gallic showed even better affinity toward Nrf2, the results of which are present in **Table 2** and **Figure 2**.

**Table 2:** Docking scores of PCT and EOT with the active sites of Nrf2 along with the interacting amino acids.

Drug	Target (PDB ID)	Interacting amino acids residues H-bond	Stacking amino acids residues pi-bond	Docking score
Piracetam	Nrf2 (2FLU)	TYR334, ARG336	ASN387, GLY386, SER383, PRO384, GLN337, ASN382	-5.079
Emblicanin A		SER508, ARG415, ARG483, SER555, SER363, ARG336, TYR572, ARG380, TYR334, ALA556,	ILE461, TYR525, HIE575, PHE577, GLY603, GLY364, GLY509	-5.182
Emblicanin B		ARG483, ASN382, ASN414, SER363, SER602, TYR334	PHE577, ALA556, TYR572, ARG380, GLY364, GLY603	-3.974
Punigluconin		ARG483, ARG380, ASN414, SER363, SER602, SER508, GLY433, CYS434, SER431, ARG415, GLY480, THR481	PHE478, ASN382, GLY603, TYR334, PHE577, ALA556, GLY509, SER555, TYR525, TYR572, GLN530, HIS436, ILE461	-6.17
Pedunculagin		GLN530, SER555, ARG415, GLY433, CYS434, SER508, HIS436, SER602, TYR525, PHE478, ARG483, ARG380,	ALA556, GLY603, SER431, PHE577, TYR334, ASN387, ILE461, ASN382, GLY509	-3.65
Gallic acid		SER363, ASN414, ARG380, ASN382	SER602, GLY603, ALA556, TYR572, PHE577, TYR334	-6.33
Rutin		GLY603, SER338, SER363, SER602, SER555, ARG415, ARG380, TYR334, ASN382	GLY364, ALA556, GLN530, TYR572, PHE478, ILE461, ASN414, PHE577	-4.66

*PDB, protein data bank*.

### Effects of EOT on Morris Water Test

**FIGURE 3:**
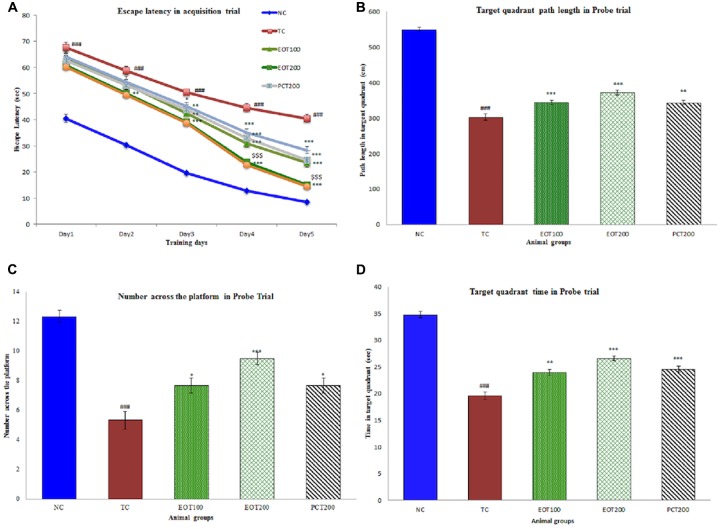
Effect of PCT and EOT on spatial learning and memory ability of rats, based on the Morris water maze test. **(A)** The escape latencies to find a hidden platform in the water maze during the five consecutive days of training. **(B)** Time spent in the target quadrant in probe task. **(C)** Number across the platform within 120 s in probe task. **(D)** The time spent in the target quadrant within 120 s in the probe task. Data are presented as mean ± SEM for six rats in each group. ^###^*p* < 0.001 vs. TC group. ^∗∗∗^*p* < 0.001, ^∗∗^*p* < 0.01, ^∗^*p* < 0.05 vs. TC group.

Positive results were obtained on the evaluation of neurobehavioral parameters using Morris water test (**Figure 3**). A decrease in escape latencies was observed in the acquisition trial. Rats in TC group had significantly greater escape latencies than normal group (^###^*p* < 0.001). Rats administered EOT200 had significantly lesser escape latencies than rats in the PCT200 group (^$$$^*p* < 0.001). Similarly, consistent results were observed when other parameters such as target quadrant path length, number across the platform in probe trial and target quadrant time in probe trial were observed. In summary, no significant difference was observed in rats administered 200 mg of tannins enriched fractions of *E. officinalis* in the Morris water test when compared to rats in the positive control group.

### Effect on GSH, GPx, MDA, and SOD Levels

In the tests of the above biomarkers of oxidative stress, it was observed that consumption of HSCD led to a significant increase in oxidative stress and that EOT supplementation led to significant attenuation of the same.

Levels of reduced glutathione (GSH) and glutathione peroxidase (GPx) were also estimated. GSH is produced in the body via the reduction of glutathione disulphide by nicotinamide adenine dinucleotide phosphate (NADPH) in the presence of glutathione reductase which acts as the catalyst (Mannervik, 2001). GSH is one of the primary antioxidants and helps in controlling oxidative damage caused by ROS. Glutathione peroxidase (GPx), on the other hand, plays an essential role in the detoxification of peroxides produced in the body (Kish et al., 1985). A significant diminution was observed in both GSH and GPx levels in the TC groups (^###^*p* < 0.001). The results obtained were consistent with previous findings. EOT administration dose-dependently ameliorated the attenuated the levels of GSH and GPx (^∗∗∗^*p* < 0.001). 200 mg dose of tannins enriched fraction of *E. officinalis* was significantly more efficacious than 200 mg piracetam in increasing GSH and GPx levels (^$$$^*p* < 0.001).

Thiobarbituric acid-reactive substances (TBARS) assay is a commonly used analytical procedure to estimate oxidative damage. The TBARS assay helps in estimating malondialdehyde, which is produced by the degradation of polyunsaturated lipids by ROS (Grotto et al., 2009). As seen in **Table 3**, a significant increase in TBARS level was observed in the TC group, which indicates oxidative stress (^###^*p* < 0.01). Moreover, it was observed that TBARS levels significantly decreased as the amount of EOT administered was increased (^∗∗∗^*p* < 0.001). Furthermore, EOT200 was found to be significantly more effective in controlling high TBARS level when compared to PCT200 (^$$$^*p* < 0.001).

**Table 3:** Effects of EOT and PCT on the activities of thiobarbituric acid reactive substance (TBARS), reduced glutathione (GSH), glutathione peroxidase (GPx) and superoxide dismutase (SOD) in the brain tissue of experimental groups.

Animal groups	TBARS (nmol/mg protein)	GSH (μmol/mg protein)	GPx (nmol NADPH oxidized/min/mg protein)	SOD (U/mg protein)
NC	2.63 ± 0.06	7.26 ± 0.09	0.284 ± 0.003	4.12 ± 0.10
TC	5.14 ± 0.15^###^	3.21 ± 0.08^###^	0.186 ± 0.004^###^	2.06 ± 0.07^###^
EOT100	4.07 ± 0.06^∗∗∗^	4.32 ± 0.08^∗∗∗^	0.237 ± 0.005^∗∗∗^	2.62 ± 0.08^∗∗∗^
EOT200	3.31 ± 0.08^∗∗∗$$$^	5.18 ± 0.06^∗∗∗$$$^	0.257 ± 0.005^∗∗∗$$$^	3.34 ± 0.07^∗∗∗$$$^
PCT200	4.32 ± 0.10^∗∗∗^	4.32 ± 0.10^∗∗∗^	0.221 ± 0.006^∗∗∗^	2.61 ± 0.08^∗∗∗^

*Data are presented as mean ± SEM for six rats in each group. ^###^*p* < 0.001 vs. TC group. ^∗∗∗^*p* < 0.001 vs. TC group. ^$$$^*p* < 0.001 vs. PCT (200 mg/kg)*.

Superoxide dismutase (SOD) catalyzes the dismutation of superoxide radicals generated as a result of oxidative stress. Hence, estimation of SOD is also an important analytical test to investigate oxidative damage. Administration of HSCD led to a significant decrease in SOD levels in the model group (^###^*p* < 0.001). This decline in SOD levels was significantly reversed in rodents administered EOT (^∗∗∗^*p* < 0.001).

### Effect on ROS Production, Mitochondrial ATP and Membrane Potential

**FIGURE 4:**
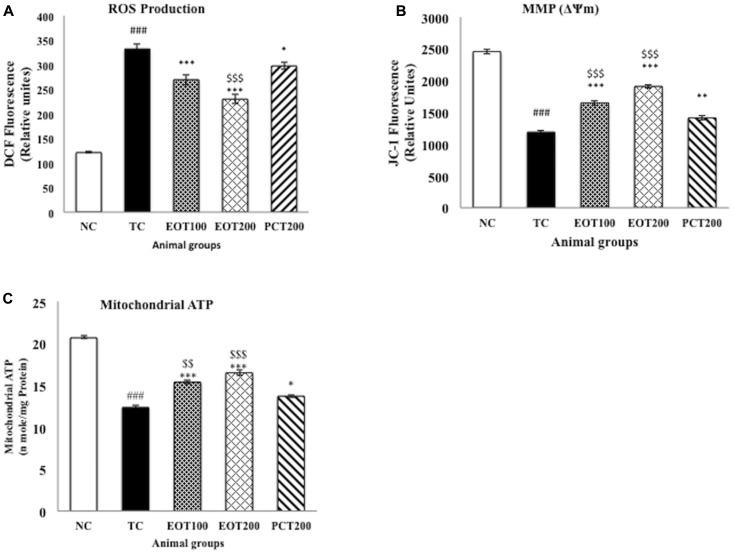
The effect of PCT and EOT treatment on the brain mitochondrial dysfunction of HSCD-fed rats. **(A)** PCT and EOT administration reduced the brain ROS production caused by HSCD consumption compared with TC group. **(B)** PCT and EOT significantly increased brain mitochondrial membrane potential change compared with TC group. **(C)** PCT and EOT significantly improved brain mitochondrial ATP compared with TC group. Data are presented as mean ± SEM for six rats in each group. ^###^*p* < 0.001 vs. TC group. ^∗∗∗^*p* < 0.001, ^∗∗^*p* < 0.01, ^∗^*p* < 0.05 vs. TC group. ^$$^*p* < 0.01, ^$^*p* < 0.05 vs. PCT200 group.

Reactive oxygen species and the resulting oxidative stress have been implicated to be a contributing factor in numerous diseases (Borroni et al., 2017; Islam, 2017). As represented by **Figure 4A**, both EOT100 and EOT200 were able to significantly reduce the increased levels of ROS observed in rats fed HSCD (^###^*p* < 0.001 for TC group vs. NC group, ^∗∗∗^*p* < 0.001 for EOT100 and EOT200 groups vs. TC group). Decrease in ROS was also observed in the group administered 200 mg of piracetam, but the diminution was relatively lesser than the EOT groups (^∗^*p* < 0.05). EOT200 group was significantly more effective in reducing ROS than the PCT200 group (^$$^*p* < 0.01).

Mitochondrial membrane potential (MMP) and mitochondrial ATP were estimated as a measure of the oxidative stress generated. **Figures 4B,C** indicates that there was a significant drop in the MMP and mitochondrial ATP in the TC group with respect to the NC group (^###^*p* < 0.001). A significant amelioration of reduced MMP and mitochondrial ATP levels was observed in EOT100 and EOT200 groups (^∗∗∗^*p* < 0.001). 200 mg piracetam was also able to significantly increase MMP and mitochondrial ATP levels (^∗∗^*p* < 0.01 and ^∗^*p* < 0.05, respectively). However, in both cases, tannoid principles of *E. officinalis* were significantly more effective in reversing the decreased MMP and mitochondrial ATP levels than piracetam.

### Effect on Brain Immunohistochemistry Analysis of Nrf2 Protein

**FIGURE 5:**
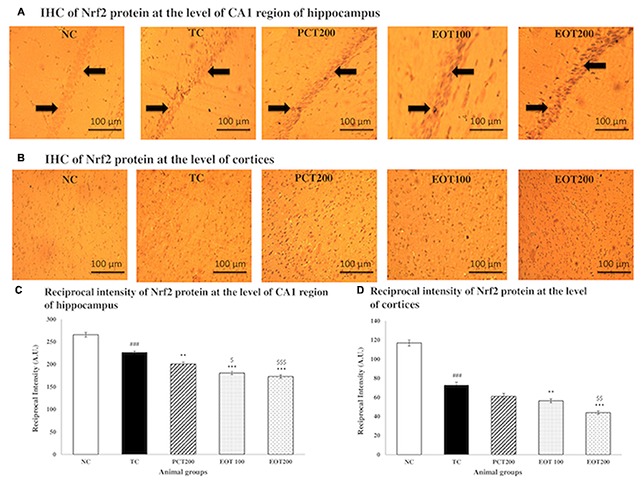
Immunohistochemistry analysis of Nrf2 protein by fluorescent microscope in coronal brain sections at the level of CA1 region of hippocampi and cortices. **(A)** Coronal brain sections at the level of CA1 region of hippocampi and **(B)** Cortices. Profound expression of nuclear Nrf2 was observed in different treatment group as compared to NC group, treatment groups of EOT and PCT have shown effect on staining of nrf2. Black arrows are showing the positively stained cells at 10× magnification. **(C)** Reciprocal intensity level of Nrf2 in the CA1 region of hippocampus. **(D)** Reciprocal intensity level of nrf2 in the cortices. Data are presented as mean ± SEM for six rats in each group. ^###^*p* < 0.001 vs. TC group. ^∗∗∗^*p* < 0.001, ^∗∗^*p* < 0.01, ^∗^*p* < 0.05 vs. TC group. ^$$$^*p* < 0.001, ^$$^*p* < 0.01, ^$^*p* < 0.05 vs. PCT200 group.

In order to further assess oxidative stress, immunohistochemical analysis was employed to evaluate Nrf2 expression in cortex tissues and the CA1 region of the hippocampi (**Figure 5**). It was observed that Nrf2 proteins were mostly present in cellular plasma, and dark brown staining was seen in the positive cells. The NC group showed low levels of Nrf2 expression in both cortices as well as the CA1 region of the hippocampi. However, in the tissue samples obtained from rats in the TC group, a significant enhancement in the Nrf2 immunoactivity was observed in both cortices as well as hippocampi. Moreover, it was observed that supplementation with EOT and administration of PCT200 in the treatment groups resulted in further increase in the expression of Nrf2. Additionally, densitometric analysis further confirmed these observations.

### Effect on Activation of Nrf2 Protein Expression by Western Blot Analysis

**FIGURE 6:**
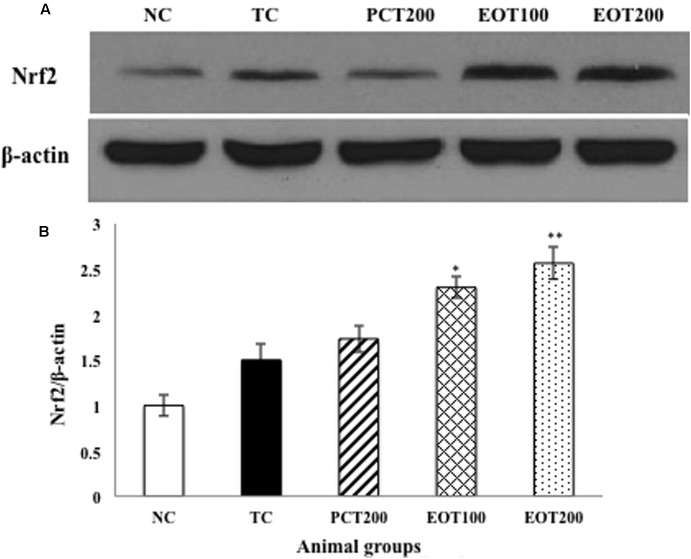
Effects of EOT and PCT on brain Nrf2 expression. **(A)** Western blot analysis of Nrf2. **(B)** Densitometry values for Nrf2 was normalized to β-actin and expressed as fold change relative to the NC group. Data are shown as mean ± SEM (*n* = 3 rats per group). ^###^*p* < 0.001 vs. TC group. ^∗∗∗^*p* < 0.001, ^∗∗^*p* < 0.01, ^∗^*p* < 0.05 vs. TC group. ^$$^*p* < 0.01, ^$^*p* < 0.05 vs. PCT200 group.

In our findings, the protein expression of Nrf2 in cytoplasmic fractions was detected to gradually decrease when treated with PCT200, EOT100, and EOT200, respectively, when compared to the TC group, indicating the translocation of Nrf2 to nucleus (**Figures 6A,B**).

### Assessment of DNA Fragmentation via TUNEL Assay

**FIGURE 7:**
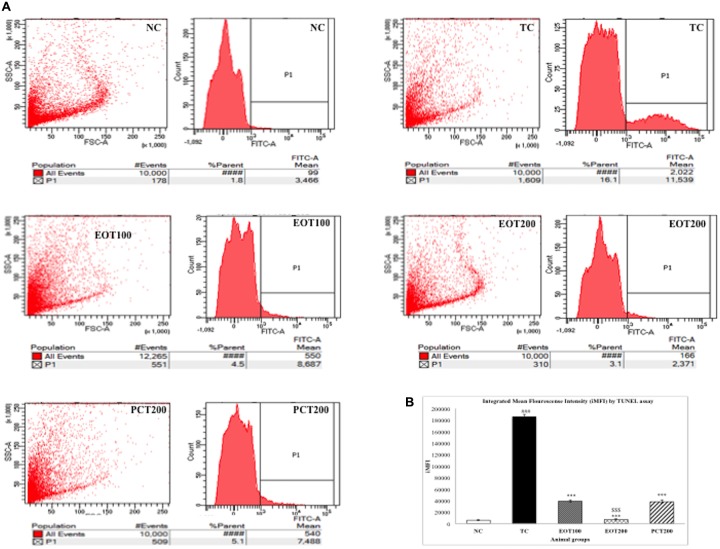
Effect of EOT and PCT on DNA Fragmentation by TUNEL assay. **(A)** The fluorescence was analyzed by flow cytometry and presented as histograms in different experimental groups. **(B)** Integrated mean fluorescence intensity (iMFI) represented as bar graphs. Data are presented as mean ± SEM for six rats in each group. ^###^*p* < 0.001 vs. TC group. ^∗∗∗^*p* < 0.001, ^∗∗^*p* < 0.01, ^∗^*p* < 0.05 vs. TC group. ^$$$^*p* < 0.001, ^$$^*p* < 0.01,^$^*p* < 0.05 vs. PCT200 group.

The terminal deoxynucleotidyl transferase [TdT]-mediated deoxyuridine triphosphate (dUTP) nick end labeling (TUNEL) assay is a commonly used analytical method to evaluate DNA fragmentation and hence, apoptosis. In the present study, flow cytometry was used to conduct the TUNEL assay. HSCD resulted in nuclear DNA fragmentation as evidenced by an increase in dUTP-FLOUS labeling. As shown in **Figure 7**, integrated mean fluorescence intensities (iMFI) significantly increased to 185777 (^###^*p* < 0.001) in the TC group, in comparison with normal control (iMFI = 6238). EOT100, EOT200, and PCT200 treated groups showed a significant reduction in iMFI levels with 39091, 7350, and 38188, respectively (^∗∗∗^*p* < 0.001). However, EOT200 was significantly more capable in controlling DNA fragmentation than PCT200 (^$$$^*p* < 0.001).

## Discussion

In the present study, we have investigated the role of HSCD in causing cognitive impairment. Moreover, we have tried to investigate the potential of supplementation by tannins enriched fractions of *E. officinalis* (EOT) to ameliorate this oxidative stress-induced cognitive impairment by their action on the Nrf2-ARE pathway.

Western dietary habits consisting of high levels of salt and cholesterol have time and again exhibited their capacity to cause cognitive impairment. Numerous epidemiological studies have shown that high levels of saturated fatty acids in the diet lead to cognitive deficits via various mechanisms such as oxidative stress, inflammation, insulin resistance, reduction in brain-derived neurotrophic factor levels, reduction in integrity of dendrites in the hippocampus (Liu et al., 2008). Obesity caused as a result of such high caloric diets has also shown to increase cerebrocortical ROS giving way for dementia (Freeman et al., 2014). In addition, presence of high quantities of salt in the diet has also been correlated with poorer brain health in humans. Hypertension, often a result of long-term diet containing high amounts of sodium, has also been implicated to cause dementia, especially in the elderly (Fiocco et al., 2012). Significantly high concentrations of salt (7–8% w/v) have been used in previous studies to induce cognitive impairment (Chugh et al., 2013; Liu et al., 2014). In the present study, the model group rats were administered 2% w/v NaCl in water as the high salt component of HSCD. It was observed that rats fed HSCD showed classical signs of cognitive impairment, which was corroborated by neurobehavioral tests (MWM) task. The 2% concentration was chosen as per previous studies investigating the potential of HSCD to induce cognitive impairment and the observations were in concordance to these studies (Mogi et al., 2007; Husain et al., 2017b).

The nuclear erythroid 2-related factor (Nrf2) is a transcription factor responsible for the regulation of expression of numerous anti-oxidative and cytoprotective genes. Nrf2 is inhibited by Kelch-like ECH-associated protein 1 (Keap1) and undergoes degradation through the ubiquitin-proteasome pathway. Exposure of cells to ROS, toxins, or electrophilic radicals leads to post-translational modification and inactivation of Keap1 which ultimately leads to attenuation of Keap1-mediated degradation of Nrf2. This causes translocation of Nrf2 proteins in the nucleus of the cells and heterodimerization of Nrf2 with small Maf proteins (sMaf), following which binding between the heterodimers and the ARE takes place in the target genes (Nguyen et al., 2009; Yamazaki et al., 2015). Through our *in silico* studies, we found that tannins enriched fractions of *E. officinalis* had good binding affinity. These *in silico* results translated well to *in vivo* studies as it was observed that EOT was able to reverse alterations in GPH, GPx, MDA, and SOD levels, which were induced due to HSCD. Increase in oxidative stress in the brain has been observed in case of mild cognitive impairment. Moreover, an elevation in oxidative stress is an established characteristic of Alzheimer’s disease (AD) and also been proposed to be used as a possible predictor for AD and other forms of dementia (Ishrat et al., 2009; Schrag et al., 2013). The central nervous system is highly vulnerable to oxidative stress due to its large consumption of oxygen, presence of sizeable quantities of free-radical generating iron and other substances like polyunsaturated fatty acids, ascorbate, glutamate, etc. which further lead to generation of radicals because of the ease by which they undergo redox-reaction (Budzynska et al., 2015). Bringing into focus the constituent tannins of EOT, both emblicanin A and B have established anti-oxidant activity (Bhattacharya et al., 1999; Bhattacharya A. et al., 2000). Furthermore, gallic acid, pedunculagin, and punigluconin have also indicated anti-oxidative potential. The docking score of each of these components, as mentioned in **Table 2**, can give us an idea about their relative effects, however, further studies are required to elucidate the individual potencies and contributions of each component in the total anti-oxidative activity of EOT (Yin et al., 2015; Yahia, 2017).

Furthermore, evaluation of neurobehavioral parameters conducted using the Morris water maze (MWM) task. The MWM task aids in the evaluation of spatial learning which is assessed through multiple, repeated trials whereas determination of reference memory is done by checking the time spent by the rodent in the quadrant where the platform was initially placed. Good spatial learning and cognition are associated with reduced escape latencies, increase in time spent in the target quadrant, as well as increase in the number of times the rodent crosses the point where the platform was placed in the acquisition trial. The results obtained from the study reflected that rats fed HSCD had poor performance in the MWM task and administration of EOT led to significantly better performance, however, there was no significant difference between the performance of the EOT groups and the PCT group. Improved performance in behavioral tests and the amelioration of cognitive deficit was consistent with previous studies evaluating the potential of EOT as well as other cognition enhancing agents (Tan et al., 2015; Thenmozhi et al., 2015).

Significant alterations in mitochondrial ATP and MMP were observed in rats fed HSCD. Accumulating evidence has shown that mitochondrial dysfunction and oxidative stress play a pivotal role in the pathophysiology of numerous neurodegenerative diseases mostly because of the fact that mitochondria have a crucial part in regulating apoptosis as well as in the aging process (Lin and Beal, 2006; Pintana et al., 2012). Multiple pre-clinical have found mitochondrial dysfunction to play a significant role in Alzheimer’s disease. Clinical studies have also revealed that mitochondrial DNA may get damaged due to oxidative stress which may ultimately lead to further neurodegeneration (Moreira et al., 2010). It has also been observed that stabilizing changes in mitochondrial ATP levels and MMP can control cell death (Richter, 1998). Through our studies, we have demonstrated for the first time that EOT supplementation was able to significantly reverse the changes in MMP and mtATP levels.

Past research has emphasized on the potential role of Nrf2 activation in controlling oxidative stress, apoptosis, and autophagy (Niture and Jaiswal, 2012; Ma, 2013). Henceforth, in order to confirm whether activation of Nrf2 was the reason behind the neuroprotective effect shown by EOT, we performed western blot, immunohistochemistry, and TUNEL assay. Western blot indicated that, when compared to the normal control group, administration of HSCD led to translocation of Nrf2 in the nucleus. Furthermore, supplementation with EOT led to a significant increase in the presence of Nrf2 in the nucleus than in the cytoplasm. Immunohistochemical analysis also showed that supplementation of EOT led to a surge in Nrf2 expression in the nucleus, which further confirmed these observations. It can clearly be observed that increase in Nrf2 expression was associated with a concomitant increase in antioxidants in the body viz. GSH, GPx, and SOD. Our results were in concordance with previous studies investigating the potential neuroprotective role of substances acting on the Nrf2-ARE pathway (Zhang et al., 2017). Nrf2 has been found to play a significant role in regulating apoptosis and studies have shown that activation of Nrf2 expression has been associated with antiapoptotic and cytoprotective actions Nrf2 has been found to suppress cellular apoptosis by regulating Bcl-2 – an apoptotic protein. A surge in the transcription of Bcl-2 leads to a reduction in Bax and cytochrome c release and activation of caspase 3/7 which consequently leads to reduced apoptosis and better cell survival (Niture and Jaiswal, 2012; Gallorini, 2015). Results obtained from the TUNEL assay indicated extensive DNA fragmentation in the model groups and also showed that supplementation with EOT led to a significant diminution in integrated mean fluorescence intensities and hence, endorsed the neuroprotective activity of EOT (Kang et al., 2015).

## Conclusion

Our results indicated that HSCD was associated with significant cognitive impairment in rats. Moreover, supplementation with tannins enriched fractions of *E. officinalis* led to significant amelioration of cognitive deficits. Our studies also indicated that activation of Nrf2-ARE pathway by EOT was the reason behind this neuroprotective effect. However, it cannot be assumed that EOT would be equally effective in humans as it was in our *in vivo* studies on rats. Hence, we believe that EOT supplementation needs to be explored further to control progression of dementia and reduce cognitive impairment. Individual efficacy of each of the EOT constituents also needs to be evaluated in order to produce a viable pharmacotherapy.

## Author Contributions

Designed the experiments: AN, IH, and MA. Performed the experiments: IH. Analysis and interpretation of data: AN, IH, and MI. Drafting the manuscript: IH, TM, and MI. Editing of manuscript: AN, MZA, and DV.

## Conflict of Interest Statement

The authors declare that the research was conducted in the absence of any commercial or financial relationships that could be construed as a potential conflict of interest.
